# Catch the Moth

**Published:** 1879-07

**Authors:** 


### CATCH THE MOTH.

Reclining at the foot of a beautiful pine whose far-reaching branches undulated gracefully in the gentle breeze that caused a low, sweet sighing, almost lulling one to slumber, we watched the awkward movements of a large moth that flew from flower to flower, sipping the honeyed dew for its morning meal.

Anxious to secure the beautiful creature for our collection, we rose from the ground, net in hand, to pursue the tortuous way of the flying insect.

So sudden was the demonstration, that a kingfisher perched on a limb above our head, took fright, and with a shrill chatter dove from the tree, dropping a minnow at our feet, that flopped and struggled until its body became enveloped with the dry, pine needles, that formed a coat of mail over its glittering sides. Picking it up, we tossed it into the stream close by, when a red squirrel took fright at the movement, scampered up the pine, and chattered among the boughs, at their utmost height, his displeasure.

Walking about the tree, looking into its top, that a glimpse might be had of the scolding animal, we felt something squirming under foot. Glancing down, we espied a beautiful green snake, ornamented with a scarlet ring about his neck, that afforded him a very becoming necklace. His beautiful black eyes sparkled like gems, his forked, scarlet tongue lapped the air, and his graceful, curving movement made him a beautiful object for contemplation. In his mouth he held a poor little frog, that had well nigh disappeared within his snakeship's interior; two slender, constantly kicking hind legs alone remaining outside.

We released the unfortunate frog from his imprisonment, when he stroked his head with each fore leg, cleared his eyes, took a survey of objects about him, winked his thanks to us, then, in three tremendous leaps, found himself secure again in his favorite pond.

The snake moved quietly, gently away into the grass, ever turning his head from side to side, protruding rapidly his tongue, making quite a demonstration of defiance as he went. Soon, came jumping from out the grass, where the snake had but just disappeared, a grasshopper, whose rapid, enormous jumps betokened his fear of the reptile. Reaching the bare ground where we stood, his jumps grew longer and carried him directly into a spider's web that hung suspended between two honeysuckle vines. Instantly, the great spider who owned the contrivance, appeared near the center of the web, then ran down to ascertain the cause of the tumultous strain upon his hawsers. He seemed to comprehend the situation of affairs at once, ran about the entangled insect in every direction, enveloping him in his tiny, silken ropes, until held a prisoner. Poor hopper! We really felt sorry for him as he lay there so helpless, while the venomous spider darted at him inflicting fatal stabs with his powerful mandibles. Before we could offer a helping hand, the victim was cruelly slain and the captor was feasting upon his body. So blood-thirsty was this act, that we confess to a sense of satisfaction when a few minutes later a king-bird, who also caught a glimpse of the performance, swooped down and carried the fat spider away, a sweet morsel, doubtless, for some young bird.

Stooping down to examine the torn spider web, we stepped upon the edge of a flat stone, which, overturning, revealed the abode of a colony of ants. What a vast family we unluckily disturbed! And how busy they at once became! What seemed to be an exploring party at once led the way to a cavity in a decayed stump. Following them were the workers who carried the worm-like young in their mouths.

By fifties and by hundreds they came, marshaled and protected on either side by their comrades, the soldiers of the expedition. In five minutes not a vestige of their former habitation was remaining,—every egg, young one, and crippled insect was safely carried to the new abode, while laborers were busy excavating and enlarging the quarters. A busier set of little creatures could not well be imagined, and rejoicing in their safety, we arose to look for our moth. We waded through the tall grass, peeped under the honeysuckle vines, searched among the blackberry blossoms, looked over the clover field, but he was nowhere to be seen. He escaped while our attention was diverted to the numerous creatures that came in our way.

When you resolve to capture a moth, pursue him until he is found fluttering in your net, and allow not yourself to be entertained by the doings of the myriads of God's creatures to be found in the woods. Reserve that capital diversion for the time when you take wife and children to camp in the country, there to enjoy the unmeasured felicity of those in love with Nature.

But, do not allow yourself to be diverted ever, from your original determination, to run into side issues. Else will your moth escape. Catch your butterfly first!

